# *Strongyloides stercoralis* and HTLV-1 coinfection in CD34^+^ cord blood stem cell humanized mice: Alteration of cytokine responses and enhancement of larval growth

**DOI:** 10.1371/journal.pntd.0009559

**Published:** 2021-07-27

**Authors:** Lauren E. Springer, John B. Patton, Tingting Zhan, Arnold B. Rabson, Hsin-Ching Lin, Tim Manser, James B. Lok, Jessica A. Hess, David Abraham

**Affiliations:** 1 Department of Microbiology and Immunology, Thomas Jefferson University, Philadelphia, Pennsylvania, United States of America; 2 Division of Biostatistics, Department of Pharmacology and Experimental Therapeutics, Thomas Jefferson University, Philadelphia, Pennsylvania, United States of America; 3 Child Health Institute of New Jersey, Robert Wood Johnson Medical School, New Brunswick, New Jersey, United States of America; 4 Department of Pathobiology, University of Pennsylvania, Philadelphia, Pennsylvania, United States of America; University of Maryland School of Medicine, UNITED STATES

## Abstract

Viral and parasitic coinfections are known to lead to both enhanced disease progression and altered disease states. HTLV-1 and *Strongyloides stercoralis* are co-endemic throughout much of their worldwide ranges resulting in a significant incidence of coinfection. Independently, HTLV-1 induces a Th1 response and *S*. *stercoralis* infection induces a Th2 response. However, coinfection with the two pathogens has been associated with the development of *S*. *stercoralis* hyperinfection and an alteration of the Th1/Th2 balance. In this study, a model of HTLV-1 and *S*. *stercoralis* coinfection in CD34^+^ umbilical cord blood hematopoietic stem cell engrafted humanized mice was established. An increased level of mortality was observed in the HTLV-1 and coinfected animals when compared to the *S*. *stercoralis* infected group. The mortality was not correlated with proviral loads or total viral RNA. Analysis of cytokine profiles showed a distinct shift towards Th1 responses in HTLV-1 infected animals, a shift towards Th2 cytokines in *S*. *stercoralis* infected animals and elevated TNF-α responses in coinfected animals. HTLV-1 infected and coinfection groups showed a significant, yet non-clonal expansion of the CD4^+^CD25^+^ T-cell population. Numbers of worms in the coinfection group did not differ from those of the *S*. *stercoralis* infected group and no autoinfective larvae were found. However, infective larvae recovered from the coinfection group showed an enhancement in growth, as was seen in mice with *S*. *stercoralis* hyperinfection caused by treatment with steroids. Humanized mice coinfected with *S*. *stercoralis* and HTLV-1 demonstrate features associated with human infection with these pathogens and provide a unique opportunity to study the interaction between these two infections *in vivo* in the context of human immune cells.

## Author summary

Human infections with the virus HTLV-1 and the nematode *Strongyloides stercoralis* are co-endemic throughout much of their worldwide ranges resulting in a significant incidence of coinfection. The coincident infections result in *S*. *stercoralis* hyperinfection and an alteration of the balance of the immune responses to the virus and to the worm. In this study, a novel model of HTLV-1 and *S*. *stercoralis* coinfection was established in humanized mice. Mortality was increased in the HTLV-1 and coinfected animals as compared to the *S*. *stercoralis* infected group. Analysis of cytokine profiles showed a distinct shift towards Th1 responses in HTLV-1 infected animals, a shift towards Th2 cytokines in *S*. *stercoralis* infected animals and elevated TNF-α responses in coinfected animals. Numbers of worms in the coinfection group did not differ from those of the *S*. *stercoralis* infected group, however, infective larvae recovered from the coinfection group showed a significant enhancement in growth. Humanized mice coinfected with *S*. *stercoralis* and HTLV-1 demonstrate features associated with human infection with these pathogens and provide a unique opportunity to study the interaction between these two infections.

## Introduction

*Strongyloides stercoralis*, an intestinal nematode parasite of humans, has an estimated global prevalence of 370 million people, mostly in tropical and subtropical regions of the world [1,2]. Humans acquire the infection via skin-penetrating third-stage infective larvae (L3i). L3i migrate to the intestine where they mature into parthenogenic female worms and release eggs that hatch into first stage larvae (L1), which continue their development either via free-living or parasitic life cycles. Most cases are asymptomatic, with eosinophilia being a primary sign of infection. Corticosteroid administration concurrent with *S*. *stercoralis* infection has been linked to the development of hyperinfection syndrome. Hyperinfection represents an amplification of the normal life cycle, leading to excessive worm burden within the usual migration pattern and the presence of autoinfective third-stage larvae (L3a) [3,4]. Symptoms can include hemoptysis, pulmonary infiltrates and frank bleeding [5] and when left untreated mortality rates may reach 87% [6,7].

HTLV-1 is a retrovirus that infects approximately 5–10 million people and is endemic in South America, Africa, Japan and the Caribbean [8]. Transmission of HTLV-1 occurs through breast feeding, sexual intercourse, transfusion of contaminated blood products or sharing of contaminated syringes and needles [9]. HTLV-1 pathogenicity is characterized by increased proliferation of CD4^+^CD25^+^CCR4^+^ T-cells [10–12]. The majority of HTLV-1 infected individuals remain asymptomatic carriers during their lives [13]. However, after a long latency period, approximately 5% of HTLV-1 carriers develop adult T-cell leukemia/lymphoma (ATLL), a CD4^+^ T-cell malignancy, and 1–4% develop HTLV-1 associated myelopathy/tropical spastic paraparesis (HAM/TSP), a chronic inflammatory disease [14]. Currently, there is no effective vaccine or therapy for HTLV-1 infection or treatment for ATLL or HAM/TSP [15].

Viral and parasite coinfections are known to lead to both enhanced disease progression and altered disease states [16,17]. Numerous sources have reported that concurrent infection with *S*. *stercoralis* and HTLV-1 is linked to the development of hyperinfection syndrome [18–21]. HTLV-1 and *S*. *stercoralis* are co-endemic throughout much of their worldwide ranges resulting in a significant incidence of coinfection. Studies show that coinfection with these pathogens can lead to increased morbidity and mortality compared to each independent infection [18–21]. HTLV-1 infection predominantly induces a Th1 response, characterized by elevated production of IFN-*γ* and TNF-α [19], whereas *S*. *stercoralis* infection predominantly induces a Th2 response, characterized by higher levels of IL-4, IL-5, IL-13 and IgE [19]. During coinfection, cross talk between the Th1 and Th2 responses can alter the immune responses within a host [22]. Some individuals coinfected with HTLV-1 and *S*. *stercoralis* have higher levels of IFN-*γ*, and lower levels of IL-4, IL-13, IL-5 and IgE than in those with only *S*. *stercoralis* infection [19]. Clinical evidence has shown coinfection results in a decrease of IL-5 and IgE responses by skewing the Th2 to a Th1 response [20]. Down-regulation of the Th2 response and a decrease in *S*. *stercoralis*-specific IgE may contribute to the severity of parasitic burden observed in hyperinfection. A decrease in IL-4 and IL-13 is associated with a reduction in numbers of L1 passed in the feces. Retention of L1 in the gut may allow them to develop into L3a. Furthermore, the decrease in IL-5 may impair the activation and proliferation of eosinophils involved in parasite killing [20]. In contrast to this, other studies have shown that coinfected patients have lower Th1 responses and increased Th2 responses. It remains unclear when one type of CD4 helper response dominates over the other in humans coinfected with HTLV-1 and *S*. *stercoralis* [23]. Patients coinfected with *S*. *stercoralis* and HTLV-1 have increased proportions of CD4^+^CD25^+^FoxP3^+^ regulatory T-cells compared to patients with either infection alone, and these proportions are inversely correlated with antigen-driven IL-5 responses. The enhanced regulatory T-cell function and the resulting reduction in the IL-5 response may explain the decreased eosinophil numbers seen in coinfected individuals [24]. *S*. *stercoralis* antigens promote clonal proliferation of HTLV-1 infected cells by activating the IL-2/IL-2R system resulting in an increased proviral load in coinfected individuals compared to asymptomatic carriers [25,26]. *S*. *stercoralis* infection is associated with increased proviral load due to both increased numbers of infected clones, as well as increased oligoclonal proliferation independent of viral integration site [14,27]. However, conflicting data have also shown a decrease in proviral load in coinfected individuals compared to asymptomatic carriers, thus the effect of *S*. *stercoralis* on HTLV-1 proviral load remains unclear [23].

Several animal models have been developed to study *S*. *stercoralis* and HTLV-1 *in vivo*. The NOD.Cg-*Prkdc*^*scid*^ *Il2rg*^*tm1Wjl*^/SzJ (NSG) mouse model can recapitulate the full spectrum of *S*. *stercoralis* infection that is observed in humans including the induction of hyperinfection in the presence of corticosteroids [4]. Balb/c-Rag1-hu^−/−^γc^−/−^ and Bone marrow Liver Thymus mice engrafted with CD34^+^ hematopoietic stem cells are susceptible to HTLV-1 infection as evidenced by detectable levels of proviral load in peripheral blood and spleen, and expression of tax protein in the brain and spinal cord [28]. NOD.Cg-*Prkdc*^*scid*^ *Il2rg*^*tm1Sug*^/JicTac (NOG) mice engrafted with human peripheral blood mononuclear cells and infected with HTLV-1 producing MT-2 cells also support infection, showing an increase of proviral load with time and oligo-proliferation of infected cells [29]. Humanized NRG mice, a phenotypically similar mouse to the NSG, have been reliably infected with HTLV-1 with polyclonal expansion of CD4^+^CD25^+^ T-cells, with leukemic T-cell overgrowth with lobulated nuclei, hypercytokinemia, and down regulation of CD3 on T-cells being observed [30]. Lastly, NSG mice, engrafted with human fetal thymus and liver and CD34^+^ stem cells isolated from the same fetal tissue one day from birth (NSG-1d), were shown to be highly susceptible to infection by HTLV-1 and demonstrated rapid polyclonal proliferation and infiltration of CD4^+^CD25^+^ T-cells to vital organs, weight loss and death [31]. Here we report the infection of CD34^+^ umbilical cord blood hematopoietic stem cell engrafted humanized NSG mice with *S*. *stercoralis*, the successful infection of such humanized NSG mice with HTLV-1, and the coinfection of *S*. *stercoralis* and HTLV-1 in these humanized NSG mice with resultant alterations in cytokine responses and parasite development.

## Materials and methods

### Ethics statement

All experimental procedures in mice were performed in compliance with the ethical and regulatory standards set by the NIH for animal experimentation. The animal use protocol (00136) was approved by the Thomas Jefferson University Institutional Animal Care and Use Committee (IACUC). This protocol adhered to the “Guide for the Care and Use of Laboratory Animals” published by the National Research Council, USA. Umbilical cord blood samples used to obtain CD34^+^ stem cells were deemed anonymous by the Thomas Jefferson University Institutional Review Board (IRB) and therefore were considered IRB exempt.

### Humanized mice

**Fig 1:**
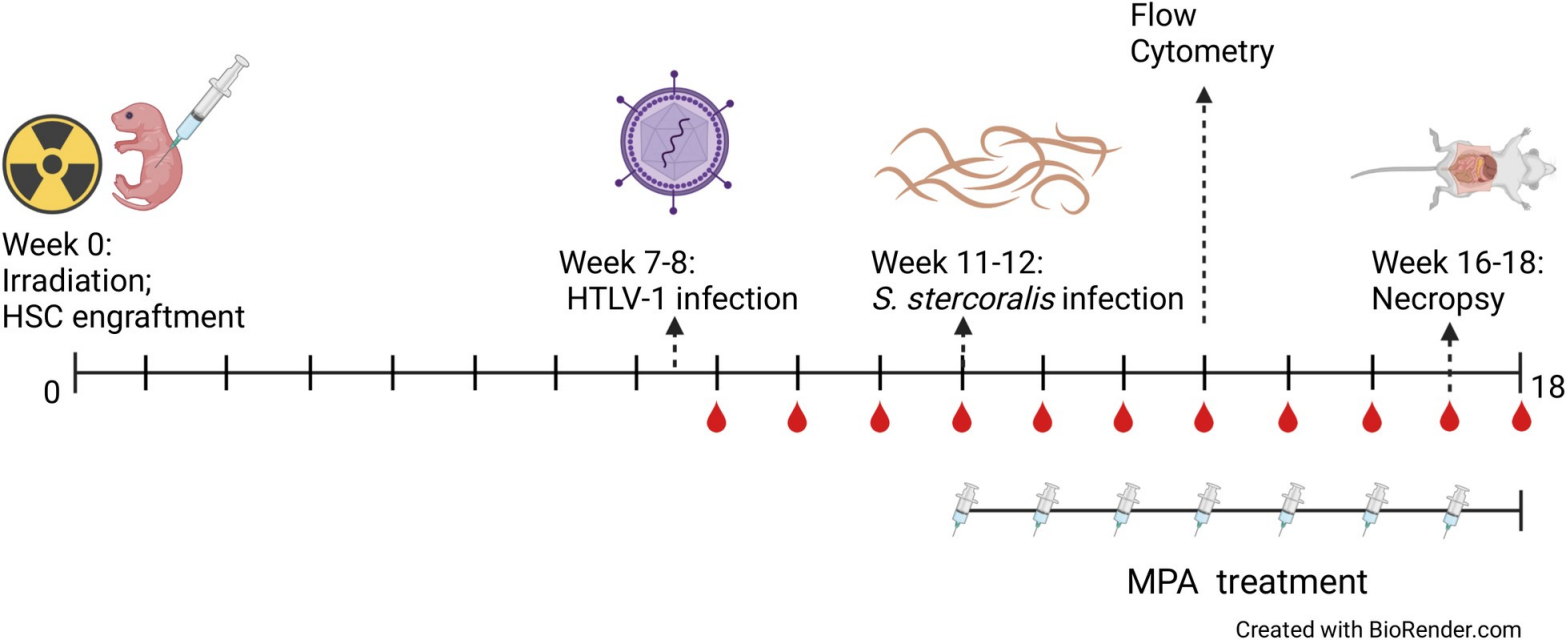
24–48-hour old NSG pups were irradiated with 1.5 Gy. Five hours post irradiation, mice were intra-hepatically injected with 10^5^ human hematopoietic stem cells (HSC) isolated from cord blood. Humanized mice were assigned to four groups: HTLV-1 infected, HTLV-1/*S*. *stercoralis* coinfected, *S*. *stercoralis* infected, and *S*. *stercoralis* infected with methylprednisolone acetate (MPA) treatment. At 7–8 weeks post HSC engraftment, coinfected and HTLV-1 infected groups received an intraperitoneal injection of 2 x 10^6^ irradiated MT-2 cells carrying HTLV-1. The coinfection, *S*. *stercoralis* infection, and MPA treated groups were inoculated with 5,000 L3i at 11–12 weeks post HSC engraftment. Steroid treated mice received 2 mg of MPA intraperitoneally weekly from the time of *S*. *stercoralis* infection until the end of the experiment. Coinfected and HTLV-1 infected groups were bled weekly post MT-2 injections and blood was analyzed for viral DNA and RNA analysis. Blood was collected from coinfected, HTLV-1 infected, and *S*. *stercoralis* infected groups once at 13–14 weeks post HSC engraftment for flow cytometry analysis. Necropsies were performed at 16–18 weeks post HSC engraftment. The figure was generated using https://biorender.com/.

NOD.Cg-Prkdc^scid^IL2rγ^tm1Wjl/SzJ^ (NSG) and C57BL/6J mice were purchased from the Jackson Laboratories (Bar Harbor, ME, USA). All mice were housed in micro-isolator boxes in a pathogen-free room at the Laboratory Animal Science Facility at Thomas Jefferson University (Philadelphia, PA). The mice were kept under temperature, humidity and light cycle controlled conditions. The NSG mouse breeding colony was sustained in-house, with breeding trios given acidified water and high fat 5K52 animal chow (LabDiet, St. Louis, MO, USA). NSG mice were humanized following previously published protocols [4,32]. Briefly, 24 to 48 hour old NSG pups were irradiated with 1.5 Gy. Five hours post irradiation, mice were intra-hepatically injected with 10^5^ human hematopoietic stem cells (HSCs), isolated from cord blood collected from full term natural deliveries (Department of Obstetrics and Gynecology, Thomas Jefferson University), in 25 μl of PBS, using a 30-gauge needle and Hamilton syringe [Fig 1]. Human CD45^+^ levels were assessed by flow cytometry. NSG mice with a minimum engraftment of 35% of the lymphocytic gate were used in the experiments.

### MT-2 cell line

HTLV-1 transformed cell line MT-2 [33] was grown in RPMI 1640 medium (Corning Cellgro, Manassas, VA, USA) supplemented with 10% fetal bovine serum (Gemini Bio-Products, West Sacramento, CA, USA), 100 U/ml penicillin, 100 μg/ml streptomycin (Corning Cellgro), and 200 nM L-glutamine (Corning Cellgro). Cells were incubated at 37°C with 5% CO_2_.

### HTLV-1 infections

HTLV-1 infected MT-2 cells were irradiated at 10 Gy and 2 x 10^6^ cells were injected intraperitoneally into humanized NSG mice [Fig 1].

### *S*. *stercoralis* L3i

UPD strain of *S*. *stercoralis* was maintained in immunosuppressed laboratory dogs at the University of Pennsylvania. Procedures on dogs were approved by and conducted in compliance with the IACUC (protocol 804883) at the University of Pennsylvania. L3i were obtained from charcoal coproculture of stools from infected laboratory dogs [34].

### *S*. *stercoralis* L3i infections

*S*. *stercoralis* L3i were concentrated by centrifugation, mixed in a 1:1 ratio with 2% low melt agarose solution (Gemini Bio, West Sacramento, CA, USA) and poured into a petri dish. Parasite-agar mixture was allowed to harden, and the L3i that migrated out of the agarose were collected and then concentrated by centrifugation in 10 ml of NI media consisting of a 1:1 mixture of NCTC-135 and Iscove’s Modified Dulbecco’s medium (Sigma Aldrich, St. Louis, MO, USA) with 100 U/ml penicillin, 100 μg/ml streptomycin, 0.25 mg/ml levofloxacin (Levaquin; Akorn, Inc., Lake Forest, IL, USA) and 0.1 mg/ml gentamicin sulfate (EMD Millipore Corp., Billerica, MA, USA). L3i were then concentrated and washed 5 times in NI media. In the initial experiments humanized mice were infected with a low dose of 500 L3i injected subcutaneously every 7 days for 4-weeks to determine parasite survival in humanized mice. In the coinfection and steroid treatment experiments, humanized mice received a single infection of 5,000 L3i at 11–12 weeks post HSC engraftment. Steroid treated mice receive 2 mg of methylprednisolone acetate (MPA; Pfizer, New York, NY, USA) intraperitoneally once per week starting from time of the *S*. *stercoralis* infection until the end of the experiment [Fig 1].

### *S*. *stercoralis* recovery

Mice were fasted for the 12 hours prior to necroscopy, anesthetized by isoflurane and exsanguinated for blood collection. Stomach, large intestines and small intestines were removed from the body cavity and were soaked in PBS to allow worms to migrate out of the tissues. The remaining tissues were minced and placed in a sieve within PBS. All tissues were incubated at 37°C, fluid was collected and concentrated by centrifugation, recovered parasites were counted and stages identified [Fig 1]. Worms were fixed with warm ethanol/glycerol (95%/5%) solution and then mounted onto slides using glycerine jelly. Parasite lengths were measured using the CellSens imaging and analysis software package (Olympus, Center Valley, PA, USA). L3i were distinguished from L3a by length and by tail morphology, with L3i having bifid tails and L3a having pointed tails [4].

### PCR

Fifty microliters of whole blood was collected retro-orbitally every week and at necropsy and placed into a tube containing 20 μl of heparin [2,000 U/ml] [Fig 1]. Erythrocytes were lysed with BD Pharm Lyse [10x] (BD Biosciences, San Jose, CA, USA). The remaining blood cells were pelleted via centrifugation and washed in PBS. Genomic DNA from the first two experimental groups was extracted from the cell pellet using Wizard Genomic DNA Purification Kit (Promega, Madison, WI, USA) following the manufacturer’s recommendations. Realtime PCR was used to determine HTLV-1 proviral levels using the SensiFAST probe Hi-ROX 2X Kit (Bioline, Taunton, MA, USA) following the manufacturer’s directions. HTLV-1 pX and human HBB gene specific primers and probes (Integrated DNA Technologies, Skokie, IL, USA) were used and human beta-globin (HBB) was used to normalize the proviral load data [HTLV-1 pX F—5’- ACAAAGTTAACCATGCTTATTATCAGC -3’, R—5’- TCTCCAAACACGTAGACTGGGT -3’, probe—5’-FAM- TTCCCAGGGTTTGGACAGAGT CTTCT -BHQ-3: Control Primers HBB F—5’- TGAGGAGAAGTCTGCCGTTAC -3’, R—5’- TGGTCTCCTTAAACCTGTCTTG -3’, Probe—5’-FAM- AAGGTGAACGTGGATGAAGTTGGTGG -BHQ-3’]. RNA from the third experimental group was extracted from pelleted blood cells using a Qiagen RNEasy kit (Qiagen, Germantown, MD, USA). The RNA samples were analyzed using the same primers as the proviral loads, using a SensiFAST probe Hi-ROX One-Step Kit (Bioline) following the manufacturer’s directions.

The TCR gene segments were amplified from peripheral blood leukocytes following previously published protocols [35]. DNA isolated from first two experimental groups were used in the clonality assay. Briefly, TCR genes were amplified with different primers designed specifically for each V segment. TCRy V2: 5’CTTC CTGCAG ATG ACT CCT ACA ACT CCA AGG TTG 3’, TCRy V3: S’CTTC CTGCAG ATG ACG TCT CCA CCG CAA GGG ATG 3’, TCRy V4: 5‘CTTC CTGCAG ATG ACT CCT ACA CCT CCA GCG TTG 3’, TCRy V5: 5’ TTC CTGCAG ATG ACG TCT CCA ACT CAA AGG ATG 3‘, TCRy V8: S‘CTTC CTGCAG ATG ACT CCT ACA ACT CCA GGG TTG 3’, TCRy V9: 5’GGNA CTGCAG GAA AGG AAT CTG GCA TTC CG 3’, TCRy V10: 5’ CT CTGCAG AAT CCG CAG CTC GAC GCA GCA 3‘, TCRy V11: 5’ CA CTGCAG GCT CAA GAT TGC TCA GGT GGG 3’, TCRy V12 (+VC): 5’ ACT CTGCAG CCT CTT GGG CAC TGC TCT AAA 3’. For the J segments the following primers were used: JGT12: 5’ AAG TGT TGT TCC ACT GCC AAA 3’, JGT3: 5’ AGT TAC TAT GAG CYT AGT CCC 3’, JGT4: 5’ TGT AAT GAT AAG CTT TGT TCC 3’. The primers were used in two separate mixes: Mix 1 (V2, 3, 4, 8, 9) and Mix 2 (V5, 10, 11, 12) all three J primers were included in each of the two mixes. The TCR segments were amplified using DreamTaq Green PCR Master Mix (ThermoFisher, Waltham, MA, USA) using PCR (45 cycles of: 95°C for 1 minute, 55°C for 1 minute, 72°C for 1 minute) and run on a 2% agarose gel with SYBR Safe DNA gel stain (1:10000) (Invitrogen, Carlsbad, CA, USA) for visualization with a UV transilluminator. The gels were analyzed for evidence of any concise bands which may indicate the presence of clonal proliferation.

### Flow cytometry

Blood was collected once, at week 13–14 (2 weeks post *S*. *stercoralis* infection) for flow cytometry analysis [Fig 1]. Whole blood was placed into tubes containing 20 μl heparin (2,000 U/ml) to prevent clotting. Erythrocytes were lysed using BD Pharm Lyse (BD Biosciences). Remaining blood cells were washed in FACS buffer [DPBS (Cellgro), 3% BSA (Gemini Bio Products), 5 mM EDTA (Sigma Aldrich) and stained in a 100-μL volume for 30 min at 4°C with the following antibodies: mouse anti-human CD45 (clone HI-30), CD4 (clone RPA-T4), CD8 (clone SK1), and CD25 (clone M-A251) (BD Biosciences). The cells were washed, fixed in 4% paraformaldehyde for 30 min, washed and then resuspended in 250 μL of FACS buffer. Cells were analyzed on a BD FacsDIVA and the data analyzed using the FlowJo X software (FlowJo, LLC, Ashland, OR). Lymphocytes were identified using forward and side scatter and human lymphocytes were identified using a human specific anti-CD45^+^ antibody.

### Luminex

Luminex cytokine assays was performed following the manufacturer’s directions on serum samples isolated prior to infection and at the terminal bleed [Fig 1]. Briefly, undiluted serum samples isolated from humanized mice infected with HTLV-1, *S*. *stercoralis*, or both HTLV-1 and *S*. *stercoralis* were analyzed using Milliplex Map Kit magnetic bead panels as per the manufacturer’s protocol (EMDMillipore). Plates were analyzed on a MAGPIX Luminex machine (Austin, TX, USA). All analyte concentrations were calculated using Milliplex Analyst software (EMDMillipore). The following 8 human cytokines were measured in the serum of the control and infected animals: IL-4, IL-5, IL-10, IL-12p40, IL-13, IL-17A, IFN-γ and TNF-α.

### Statistics

All experiments consisted of 3–9 mice per group and experiments were performed three independent times. Data presented are collected from all experiments except for proviral load results. Kaplan-Meier curves of the four groups as well as the group-wise log-rank test were analyzed in Prism 7.0 (Systat Inc., Evanstown, IL, USA) and R [36]. Parasite recovery, T-cell populations, and worm length data were analyzed by multifactorial analysis of variance ANOVA with post-hoc Fisher’s Least Significant Difference (LSD) testing in Systat v.11 (Systat Inc.) and R (Vienna, Austria). *P* < 0.05 was considered statistically significant. For each cytokine measurement, a non-detectable result, resulting in a ‘0’ signal strength was replaced by ‘1’ in the analysis so that the log-transformation on signal strength could be performed. The effect of infection on cytokine responses was assessed by comparing week-7 levels to Day 0 baseline, which was regarded as offset. Evaluation was performed using generalized linear regression models on the log-transformed cytokine signals allowing for within-group heteroscedasticity structure if adequate. The generalized linear regression models, as well as the group-wise comparisons (without multiple test adjustment), were fitted using R packages nlme and multcomp [37,38].

## Results

### Humanized-mouse response to *Strongyloides stercoralis* infection

**Fig 2:**
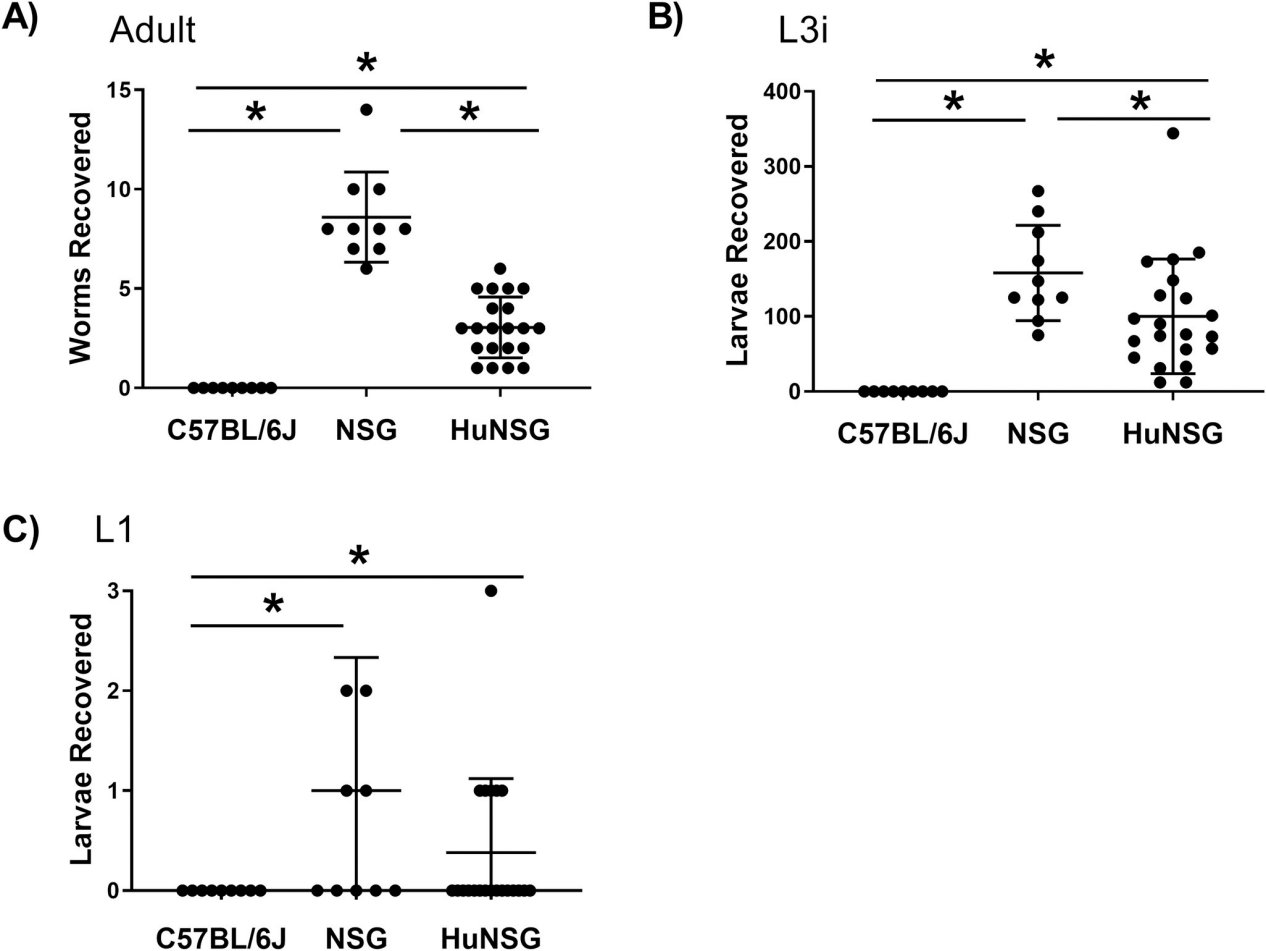
C57BL/6J, NSG and humanized NSG mice were infected with 500 infective third-stage larvae (L3i) of *Strongyloides stercoralis* weekly for 4-weeks to determine susceptibility of the mice to the infection. Adults (A), L3i (B) and first stage larvae (L1) (C) were collected and counted. Data presented are means ± standard deviations and asterisk represents p < 0.05.

Humanized NSG mice were infected with 500 L3i weekly for 4-weeks to determine if the mice were susceptible to infection with *S*. *stercoralis* and if their human immune system mounted a response to the infection following repeated exposure. At 6-weeks post final infection, the humanized mice were dissected to recover parasites from the tissues. There were significantly fewer adults [Fig 2A] and L3i [Fig 2B] recovered from the tissues of infected humanized mice than NSG mice. No significant differences were seen in the numbers of L1 [Fig 2C] recovered from the infected NSG mice and humanized NSG mice. CD34^+^ humanized mice were found to support all stages of the *S*. *stercoralis* lifecycle, although with lower numbers of parasites than seen in NSG mice.

### Mortality rates of humanized mice infected with *S*. *stercoralis* and/or HTLV-1

**Fig 3:**
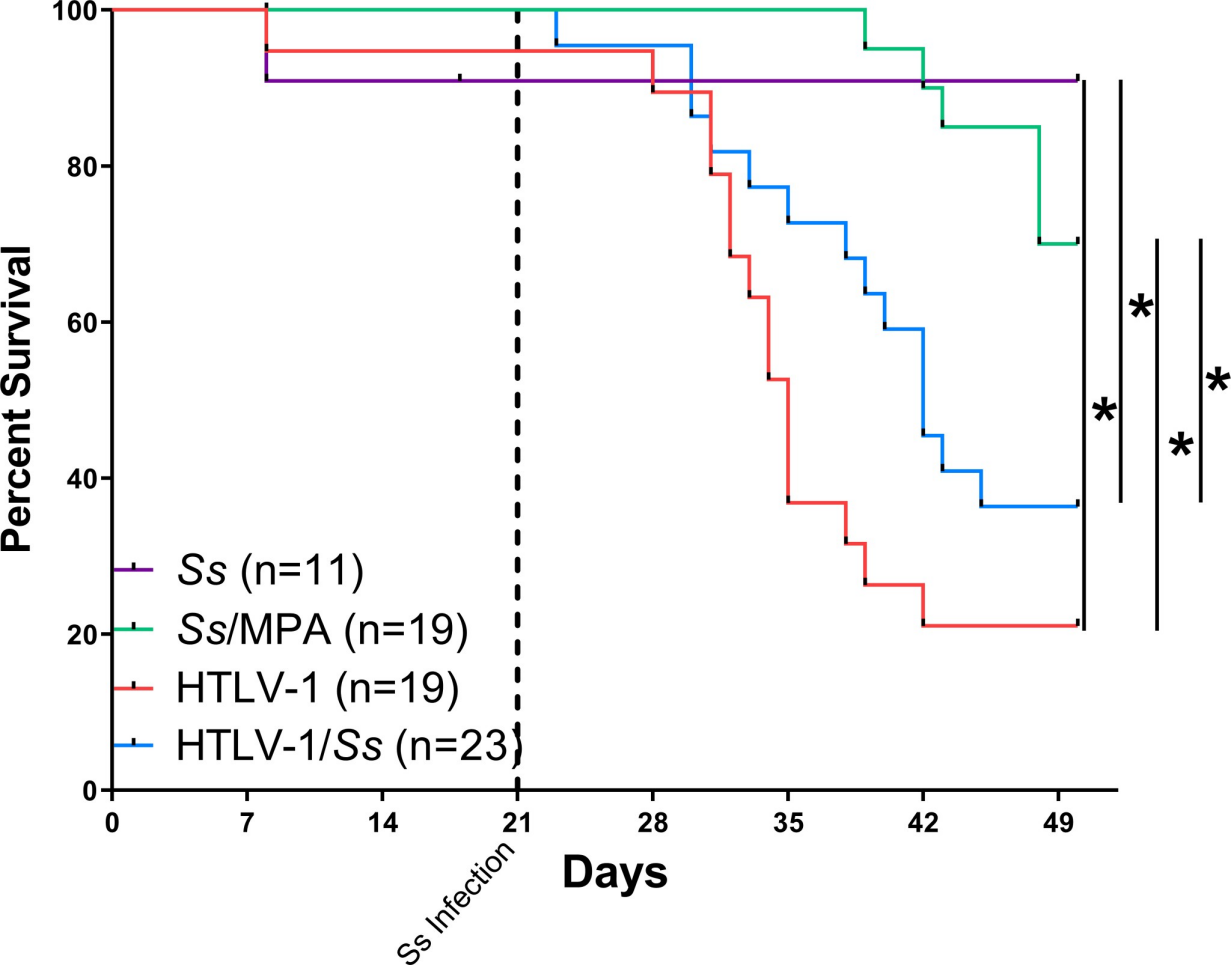
Mortality rates of humanized NSG mice during infection with HTLV-1 and *Strongyloides stercoralis (Ss)*. Mice were infected with either 5,000 *S*. *stercoralis* infective third-stage larvae, HTLV-1 or a combination of the two pathogens. As a positive control group, mice infected with *S*. *stercoralis* were treated with the steroid methylprednisolone acetate (MPA). Mortality rates were tracked throughout the 7-week course of the infection. The dotted vertical line indicates the date the mice were infected with *S*. *stercoralis*, 21 days post infection with HTLV-1. Mortality rates are a combination of three independent replicates and the total n value for each experimental group is included in the figure. Asterisk represents p < 0.05.

Previous studies using NSG mice demonstrated that injection of 5,000 L3i into the mice resulted in consistent and well tolerated infections [4]. Humanized mice infected with 5,000 L3i of *S*. *stercoralis* tolerated the infection well with 91% of infected mice surviving for at least 7- weeks. Humanized mice infected with *S*. *stercoralis* and treated with MPA to induce hyperinfection had 70% survival at 7-weeks. This is in contrast to humanized mice infected with HTLV-1, which had only 21% survival, while coinfected humanized mice had survival of 34% at 7-weeks. The 13 percent decrease in the survival observed when comparing HTLV-1 and coinfected animals [p = 0.07] suggests an interplay between the two pathogens affecting the viability of the infected host [Fig 3].

### HTLV-1 proviral and total viral RNA levels

**Fig 4:**
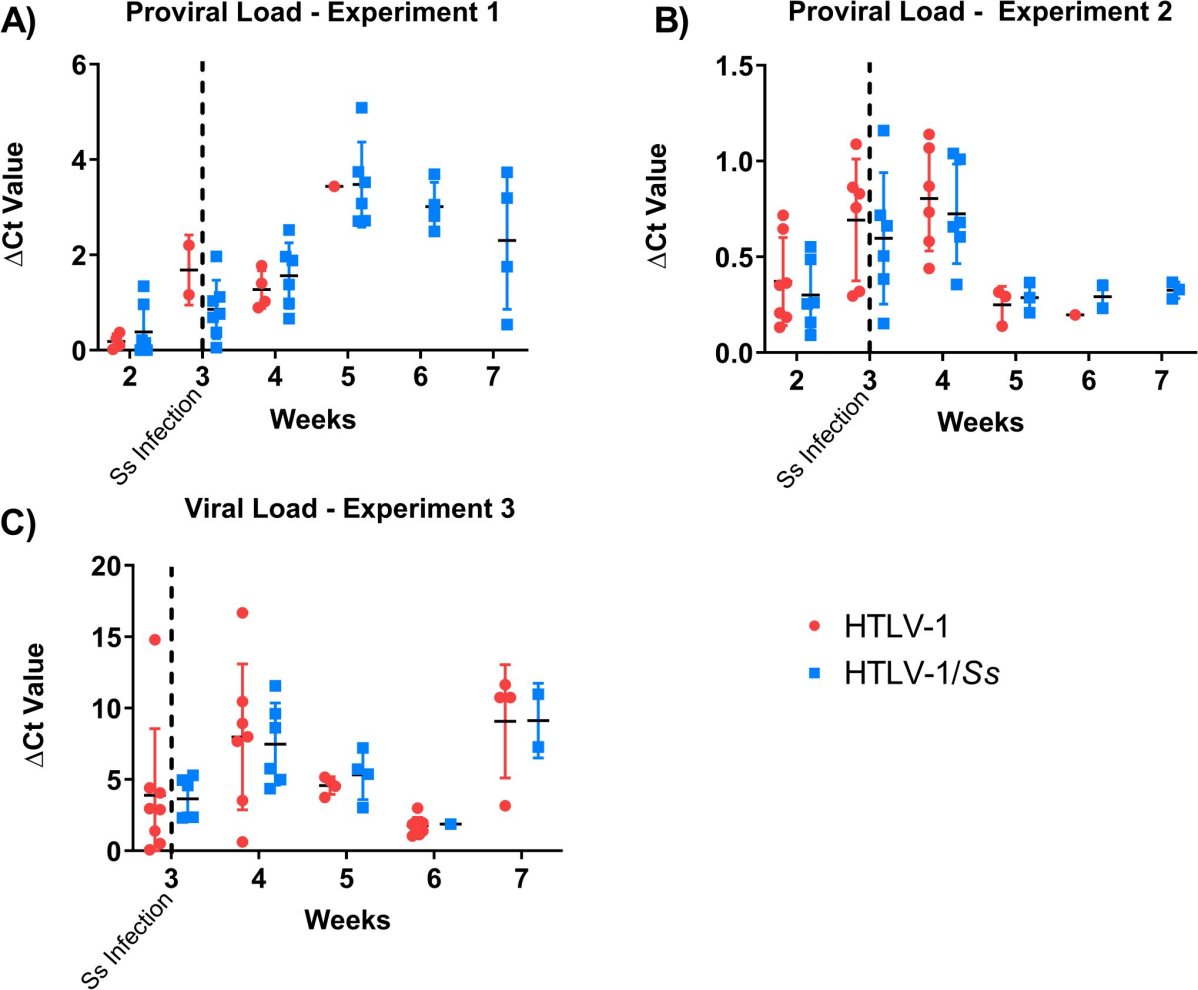
Proviral and viral load levels in humanized NSG mice during infection with HTLV-1 and *Strongyloides stercoralis (Ss)*. Proviral loads in white blood cells were determined using real-time PCR with primers and probes specific for the HTLV-1 pX region. The HTLV-1 total RNA levels in the white blood cells were determined using real-time PCR. Results for proviral load measurements from two independent experiments are presented (A & B) and viral RNA levels from one experiment (C). No significant difference was observed between HTLV-1 infected and coinfected animals. The vertical dotted line represents time of *S*. *stercoralis* infection with 5,000 third stage larvae.

HTLV-1 proviral loads and total viral RNA levels were measured to assess the relationship between HTLV-1 infection and the enhanced mortality observed in humanized mice. Proviral loads were determined in human PBMC isolated from retro-orbital blood of humanized mice using primers specific to the pX region in real time PCR assays. The total proviral load in human PBMC increased during the first 4-weeks of infection in two independent replicate experiments. From week-5 through 7 two separate patterns were observed. In one cohort of animals the proviral loads continued to increase in week-5, sustaining these levels through weeks 6 and 7 [p <0.001] [Fig 4A], while in the other, the proviral loads significantly decreased in weeks 5–7 from their 4-week high [p < 0.01] [Fig 4B]. Total viral RNA levels, measured from weeks 3 to 7, increased from week 3 to 4 following a similar trend to that found for proviral loads. Unlike proviral DNA levels in the previous two experiments, a third pattern emerged with viral RNA levels decreasing after week-4 and then rebounding at week-7 [Fig 4C]. No significant correlations were observed between mortality of infected mice and HTLV-1 proviral load or total RNA levels, suggesting that these levels did not play a direct role in the elevated mouse mortality rates.

### Human cytokines measured in the serum of infected humanized mice

**Fig 5:**
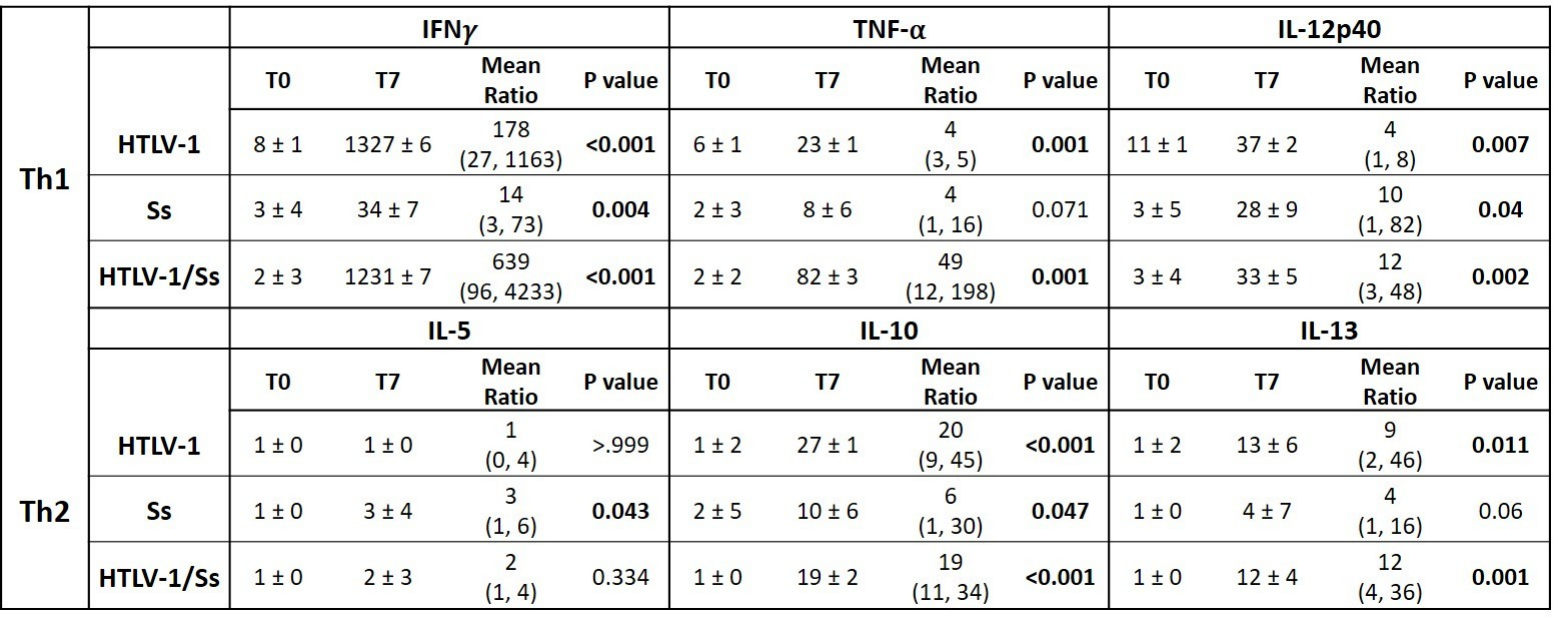
Comparison of human Th1 and Th2 cytokine levels measured in serum from mice infected with HTLV-1, *Strongyloides stercoralis* (*Ss*) or coinfected with both HTLV-1 and S. stercoralis. Measurements were taken of preinfection sera (T0) and at 7-weeks post infection (T7). Geometric mean and standard deviations of cytokine levels are presented. The estimated mean ratios as well as 95% confidence intervals are presented with P values. For clarity of presentation all numbers are rounded without decimal points.

HTLV-1 infections in humans are associated with Th1 CD4^+^ cell cytokine responses [39, 40] while *S*. *stercoralis* infections often present with Th2 cytokine responses [41]. Serum was recovered from infected humanized mice and the human cytokines IFN-γ, TNF-α and IL-12p40 were measured to monitor Th1 responses, and IL-4, IL-5, IL-10 and IL-13 measured to monitor Th2 responses, and IL-17A measured to monitor Th17 responses. At 7-weeks post-infection the human cytokines IFN-γ, TNF-α, IL-12p40, IL-10 and IL-13 were elevated in humanized mice infected with HTLV-1, *S*. *stercoralis* or with the dual infection, as compared to background controls. IL-5 was significantly elevated only in humanized mice infected with *S*. *stercoralis*. IL-4 and IL-17A levels were not significantly elevated, as compared to background, in any of the infected humanized mice. The levels of the cytokine responses differed greatly, with IFN-γ responses in HTLV-1 infected mice 178 times higher than baseline whereas IL-5 levels in *S*. *stercoralis* infected mice were elevated 3 times over baseline [Fig 5].

Group-wise comparison of the 7-week infection-induced cytokine responses, regarding the day 0 baseline responses as an offset, were performed. Significant differences were only noted in IFN-γ and TNF-α responses between infection groups at week-7. The IFN-γ levels in the *S*. *stercoralis* group was only 2% of the dual infection group [95% CI 0.00~0.22, p < 0.001], and only 8% of that of the HTLV group [95% CI 0.01~0.79, p = 0.031]. There was no significant difference in the IFN-γ levels between the dual infection group and the HTLV-1 group. On the other hand, for TNF-α the levels in the HTLV group was 8% of that of the dual infection group [95% CI 0.02~0.29, p< 0.001] and the levels of the *S*. *stercoralis* group was 8% of that of the dual infection group [95% CI 0.01~0.49, p = 0.006]. Thus, differences in the cytokine levels between the groups of infected mice at 7-weeks post-infection showed humanized mice infected with HTLV-1 or coinfected with HTLV-1 and *S*. *stercoralis* had significantly higher IFN-γ levels than that seen in mice only infected with *S*. *stercoralis*. TNF-α levels were significantly enhanced in coinfected animals when compared to levels in mice infected with only *S*. *stercoralis* or only HTLV-1.

### Analysis of the human CD4^+^CD25^+^ T-cells population in HTLV-1 and HTLV-1/*S*. *stercoralis* infected humanized mice

**Fig 6:**
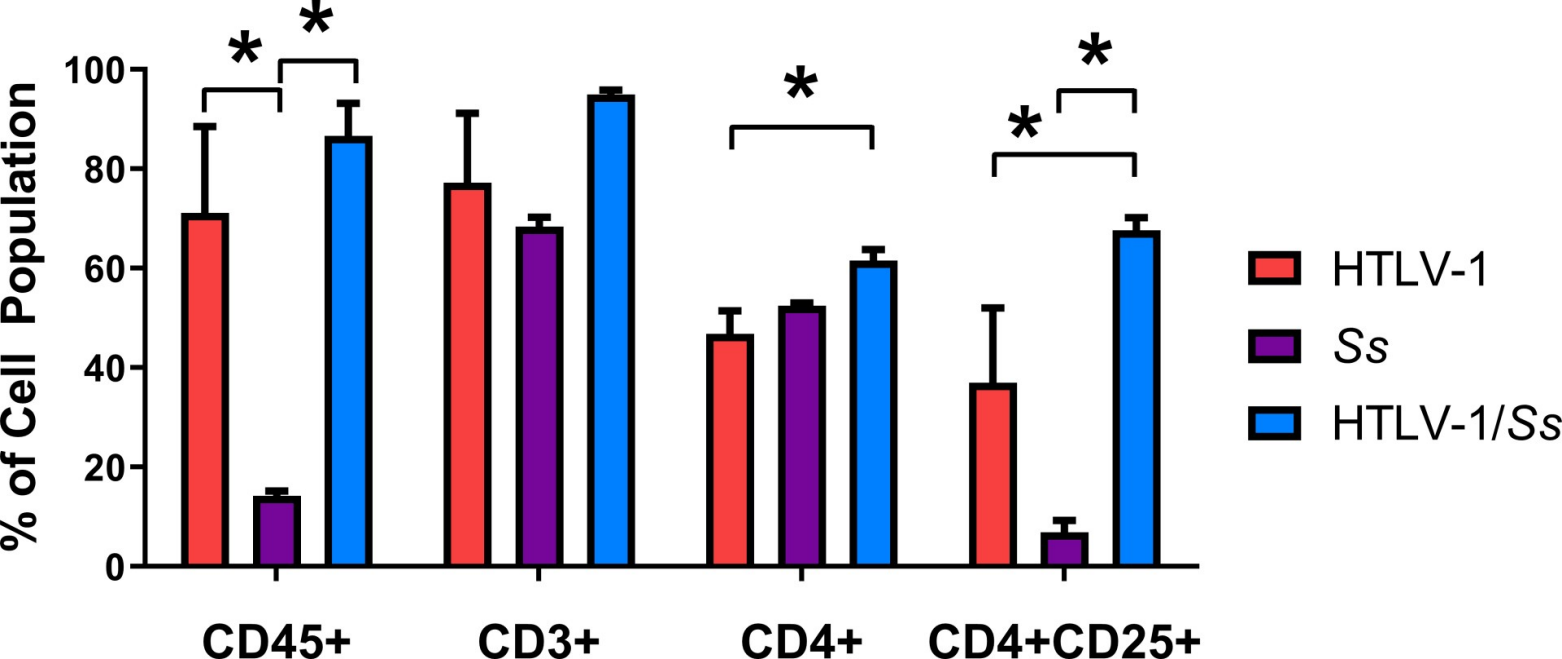
Composition of the human immune cells from peripheral blood in humanized NSG mice during HTLV-1 and *Strongyloides stercoralis (Ss) co*infection taken at 2-weeks post-infection with *S*. *stercoralis*. Human lymphocytes were stained with mouse anti-human CD45, CD3, CD4, CD8, and CD25 and analyzed by flow cytometry. Asterisk represents p < 0.05.

Flow cytometry was performed on human PBMC isolated from blood recovered from infected humanized mice to determine the changes in T-cell subsets associated with infection with HTLV-1 and/or *S*. *stercoralis*. This analysis was performed at only one time point because of restrictions in blood volumes that could be obtained from individual mice. The cohorts of mice used in this analysis were comprised of humanized mice generated from multiple stem cell donors in an effort to diminish any donor bias and thus ensure that representative immune responses would be observed. At 2-weeks post infection with *S*. *stercoralis*, the CD45^+^ lymphocyte population in humanized mice was 14% ± 1.4 of the lymphocyte gate and granulocyte gate based on forward and side scatter, which was statistically lower than the 71% ± 35 in HTLV-1 infected and 87% ± 15 in HTLV-1/*S*. *stercoralis* coinfected animals. While no significant differences were observed in the total number of CD3^+^ cells at this time point, the number of CD4^+^ cells was significantly higher in HTLV-1/*S*. *stercoralis* coinfected mice as compared to mice infected with only HTLV-1. CD4^+^CD25^+^ T-cell populations were significantly elevated in HTLV-1/*S*. *stercoralis* coinfected mice [68% ± 6] as compared to HTLV-1 infected mice [37% ± 30] [Fig 6].

### Clonality of TCR

Population clonality was examined by PCR to determine if the enhanced population of CD4^+^CD25^+^ cells represented a clonal proliferation possibly due to ATLL. The amplified variable regions of the T-cells isolated from the mouse peripheral blood were analyzed by gel electrophoresis for evidence of concise bands which may indicate the presence of clonal proliferation. In the absence of any concise bands, it was concluded that there was general T-cell proliferation and not clonal proliferation of these T-cells.

### *S*. *stercoralis* stages recovered from humanized NSG mice

**Fig 7:**
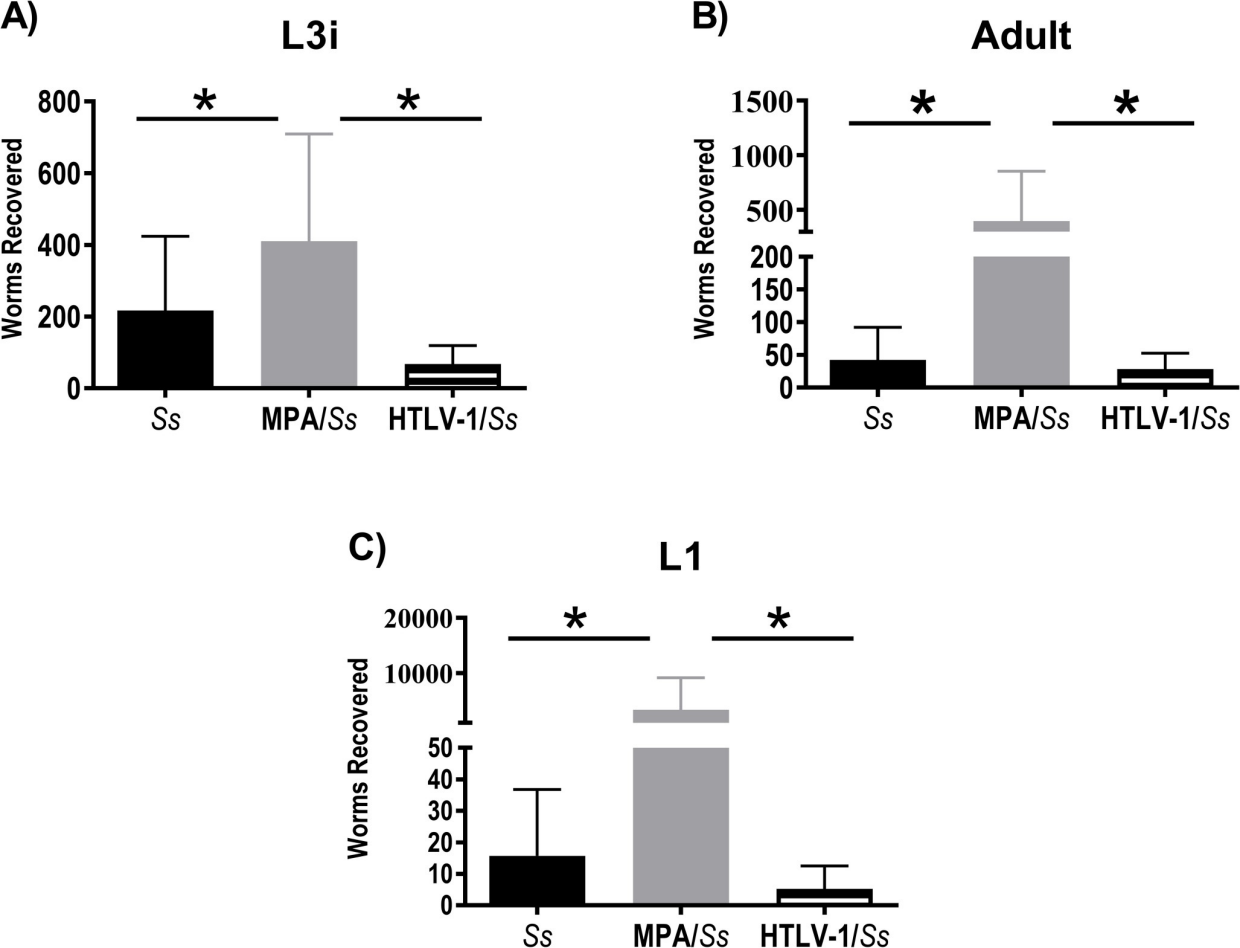
Effects of HTLV-1 infection and methylprednisolone acetate (MPA) on *Strongyloides stercoralis* infection in humanized NSG mice 7-weeks post-infection. Mice were infected with 5,000 *S*. *stercoralis* infective third-stage larvae (L3i) and either treated with MPA or infected with HTLV-1. Data presented are means ± standard deviations and asterisk represents p < 0.05 for numbers of (A) L3i, (B) adults and (C) first stage larvae (L1) recovered from the mice.

Parasites were recovered 4-weeks post-infection from humanized mice to determine if coinfection with HTLV-1 altered the developmental profile of *S*. *stercoralis*. Parasites were categorized as L3i, adult or L1 [Fig 7A–7C]. No significant differences were seen in L3i, adult or L1 numbers in the *S*. *stercoralis*/HTLV-1 coinfected mice as compared to the *S*. *stercoralis* infected mice. Significantly increased numbers of L3i, adult worms and L1 were seen in MPA treated humanized-mice infected with *S*. *stercoralis* as compared to untreated *S*. *stercoralis* infected mice and in mice with coinfections [Fig 7A–7C].

**Fig 8:**
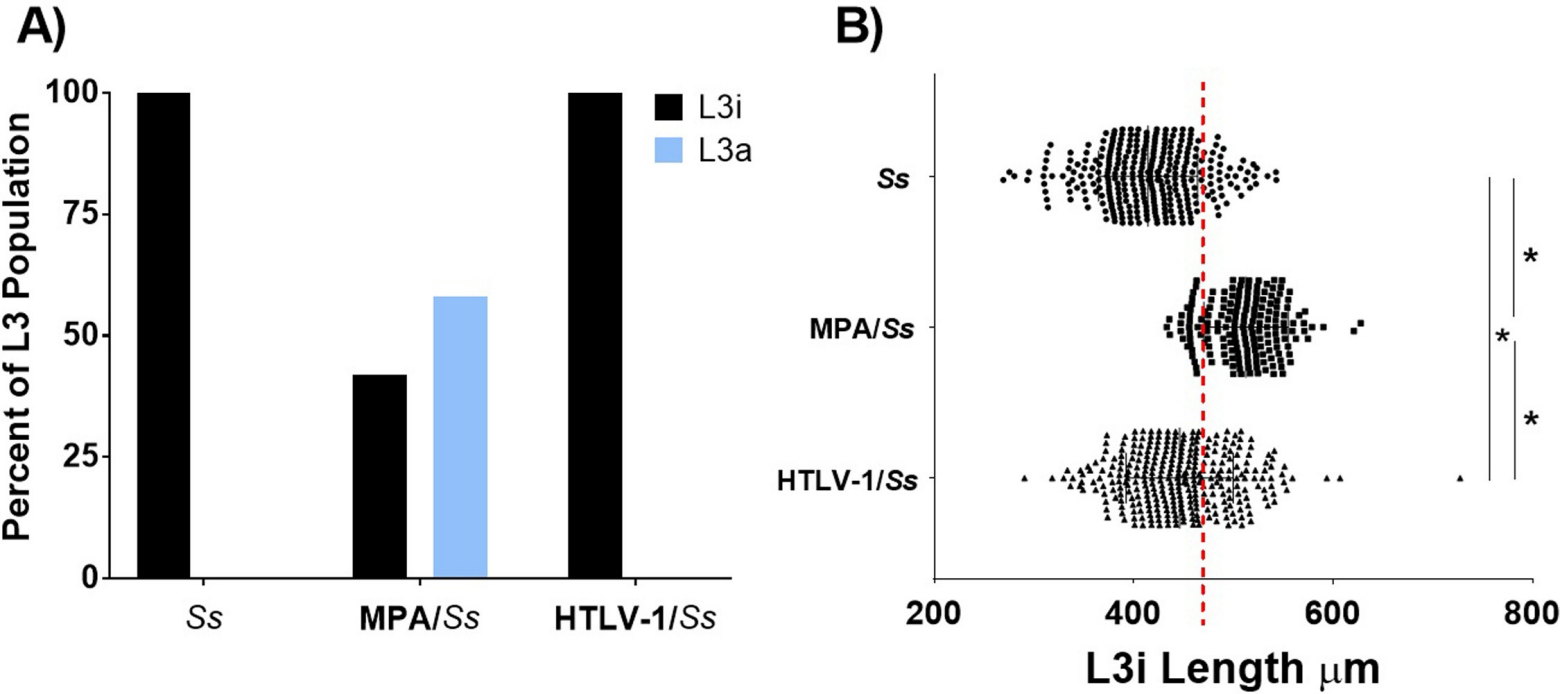
Effects of infection with HTLV-1 or treatment with methylprednisolone acetate (MPA) on the development of *Strongyloides stercoralis* autoinfective third stage larvae (L3a) and lengths of third-stage larvae (L3i) recovered from humanized NSG mice. Mice were infected with 5,000 *S*. *stercoralis* L3i and either treated with MPA or infected with HTLV-1. (A) Percentage of L3i and L3a recovered from the body tissues of *S*. *stercoralis* infected animals. (B) Lengths of L3i recovered from the body tissues of *S*. *stercoralis* infected humanized NSG mice. Data are a combination of three independent replicate experiments. Asterisk represents p < 0.05. Red dashed line marks 470 μm.

One of the hallmarks of *S*. *stercoralis* hyperinfection is the presence of L3a in the tissues of infected animals [4]. Only MPA treated humanized mice infected with *S*. *stercoralis* had L3a isolated from their body tissues, based on both tail morphology and length [Fig 8A]. Measurement of the recovered L3i, however, revealed that the L3i isolated from the coinfected mice were significantly longer [446 μm ± 54] than those isolated from animals only infected with *S*. *stercoralis* [415 μm ± 50]. L3i isolated from humanized-mice with hyperinfection caused by MPA were significantly longer [513 μm ± 42] than L3i recovered from untreated mice infected with *S*. *stercoralis* and mice coinfected with both *S*. *stercoralis* and HTLV-1 [Fig 8B]. This observation was confirmed in three replicate experiments. There appeared to be two distinct populations of L3i recovered from mice coinfected with *S*. *stercoralis* and HTLV-1, with 30% of L3i larger than 470 μm and 70% smaller. Using the same criteria, 87% of the L3i recovered from mice infected with only *S*. *stercoralis* were in the smaller group and 84% of the L3i recovered from mice coinfected with *S*. *stercoralis* and HTLV-1 were in the larger group. The larger L3i recovered from mice coinfected with *S*. *stercoralis* and HTLV-1 had a mean length of 509 μm ± 35 that was statistically equal to the L3i recovered from mice treated with MPA and different from L3i recovered from untreated humanized mice [p < 0.001]. The smaller L3i recovered from mice coinfected with *S*. *stercoralis* and HTLV-1 had a mean length of 419 μm ± 34 that was statistically equal to the L3i recovered from untreated humanized mice and different from L3i recovered from mice treated with MPA [p < 0.001]. It was therefore concluded that both MPA and coinfection with HTLV-1 enhance the growth of *S*. *stercoralis* L3i in humanized mice.

## Discussion

HTLV-1 and *S*. *stercoralis* coinfection has been associated with the development of *S*. *stercoralis* hyperinfection [18–20,42,43], a disease with high mortality rates when left untreated [42,44]. Small animal models utilizing NSG mice have been described for both HTLV-1 and for *S*. *stercoralis* hyperinfection [4,30,31]. In the present study, the first dual infection model for HTLV-1 and *S*. *stercoralis* is described, utilizing NSG mice that have been humanized with human hematopoietic stem cells [32,45]. Infection of humanized mice with HTLV-1 and/or *S*. *stercoralis* resulted in multiple facets of the clinical etiology that match with human disease progression, including variability within the proviral and total viral RNA loads of HTLV-1 depending upon host, strong Th1 cytokine responses to HTLV-1 infection, limited Th2 cytokine responses to *S*. *stercoralis* infection, mortality of humanized mice with *S*. *stercoralis* hyperinfection and enhanced development of the parasite in the coinfection model when compared to a solitary *S*. *stercoralis* infection [42,43,46,47]. This novel model will allow researchers, for the first time, to experimentally observe the interplay between these two pathogens.

The human immune system present in the CD34^+^ humanized mice has functional elements as well as multiple deficiencies in immune components. The stem cells are capable of differentiation into an immature, partially functional human immune system consisting of human B-cells, T-cells, monocytes and dendritic cells. Although, the functionality of each of these cell types is often compromised, they do recapitulate many of the functions of the human immune system [32, 45]. Humanized mice were infected with 4 low doses of *S*. *stercoralis* to determine if these mice were susceptible to infection and if the human immune system was capable of recognizing and controlling the infection. All stages of the *S*. *stercoralis* life cycle developed in humanized mice. There was, however, a significant decline in the number of L3i and adult worms that were recovered from the humanized mice as compared to NSG mice. These findings demonstrate that humanized mice are susceptible to infection with *S*. *stercoralis* and based on the decrease in numbers of L3i and adult worms recovered from humanized mice, it appears that the transplanted human immune system may play a role in controlling the repeated infection with L3i of *S*. *stercoralis*.

Humanized mice tolerated infection with *S*. *stercoralis* with minimal mortality, closely approximating rates seen in humans during uncomplicated *S*. *stercoralis* infections. When humanized mice were infected with HTLV-1 alone, a 90% mortality rate was observed by week-7. Premature death was also found to be a limitation in the NSG-d1 model, in which premature death was related to the incomplete reconstitution of a normal immune system, and the rapid polyclonal proliferation of CD4^+^CD25^+^ T-cells that infiltrate organs causing weight loss and death [31]. Interestingly, HTLV and *S*. *stercoralis* coinfected mice exhibited longer survival as compared with HTLV-1-infected mice alone in two of three separate experiments using different donor CD34^+^ stem cells, with an overall tendency for slower progression and increased survival by 49 weeks [Fig 3]. These data are reminiscent of a surprising clinical observation that coinfection with *S*. *stercoralis* in patients with HTLV-1 associated ATLL was associated with prolonged survival as compared with ATLL patients not infected with *S*. *stercoralis* [48]. Thus, this novel coinfection model may provide an avenue to begin to address this surprising clinical feature of ATLL. In the present study clonal proliferation of the CD4^+^CD25^+^ T-cells was not detected however, a general proliferation was observed. While the typical rate of mortality in humans due to early and uncomplicated HTLV-1 infection is low, a much higher rate was observed in the humanized mice. Mortality due to HTLV-1 infection is typically caused by the progression of infection particularly to ATLL, although increased mortality is observed associated with HAM-TSP, and even in asymptomatic infection [49]. While ATLL and HAM/TSP only happen in a small percentage of HTLV-1 infected patients, they carry a significant mortality rate [9,10,50]. The failure to observe a clonal ATLL-like phenotype in the mice was likely due to rapid progressive disease caused by large-scale CD4^+^ T-cell population with increased levels of cytokine production, eliminating the time needed for progressive clonal expansion associated with additional genomic mutations, as seen in ATLL [51].

The mortality rates for the MPA-treated *S*. *stercoralis-*infected humanized mice reached 30% at 4-weeks post-parasite infection, similar to the mortality rates seen in infected MPA treated NSG mice [4]. Previous studies have shown that the mortality rates increase over time in NSG mice with MPA induced hyperinfection and correlated with dissemination of the parasites throughout the host, septicemia and significantly enhanced worm burden [4]. Due to the high mortality rates of the HTLV-1 infected cohorts, experiments in this study were limited to 4-weeks post-parasite infection in order to ensure a statistically significant population of viable HTLV-1 infected mice.

The overall levels of proviral DNA and total viral RNA in circulating human PBMC in humanized mice were measured to investigate a possible cause for mortality in mice infected with HTLV-1. Similar to the CD133^+^ humanized mice [30], the proviral load increased for the first 4-weeks of infection, a trend that was also mirrored by the total viral RNA levels. After the initial 4-weeks of infection, infected mice from the three replicate experiments displayed three different patterns of proviral loads and total viral RNA. In one case the proviral load continued to increase consistent with the previously reported finding [30], in another, the proviral load dropped and then stabilized and in the third experiment the total viral RNA dropped at week-5 only to rebound the following week. Various studies have examined HTLV-1 proviral loads to determine if they can be used as indicators of future disease development and progression in asymptomatic carriers of HTLV-1. Proviral loads vary within HTLV-1 asymptomatic carriers and patients with HTLV-1 related diseases [14,27,52]. Individuals who developed HAM/TSP or ATLL presented with a persistently higher viral load than those who remained asymptomatic [47,53–55]. In addition, within a single host, proviral load often remains stable, but can vary by more than 1,000-fold among hosts [14]. Multiple donors of CD34^+^ HSCs were used to engraft the groups of humanized mice used in the present study. This may explain the variability seen in levels of proviral DNA and total viral RNA seen between the three experiments. Regardless, proviral load does not appear to be associated with mortality in this model, as HTLV-1 proviral loads continued to increase in the surviving animals.

Coinfection of people with HTLV-1 and *S*. *stercoralis* results in increased HTLV-1 proviral load, clonal abundance and oligoclonal expansion of T-cells [14,27,52]. *S*. *stercoralis* soluble antigen stimulates the oligoclonal proliferation of HTLV-1 infected CD4^+^ T-cells in HTLV-1 asymptomatic carriers, with proviral load being up to five times higher in HTLV-1 carriers coinfected with *S*. *stercoralis* than in those infected with HTLV-1 alone [26]. Contrary to this finding, no difference in proviral loads was seen between HTLV-1 infected mice and the *S*. *stercoralis*/HTLV-1 coinfected mice. It is possible that an interaction between HTLV-1 and *S*. *stercoralis* resulting in increased viral load would have been observed in humanized mice if the infections were allowed to persist in them for extended time periods, instead of being limited by the early lethality of the HTLV-1 infection.

HTLV-1 infection causes an enhancement in patient’s Th1 responses that includes increases in a number of cytokines [10,39,56] and studies have shown that infection with *S*. *stercoralis* skews the immune response towards Th2 responses [20]. Human cytokine levels were measured in the serum of humanized-mice infected with *S*. *stercoralis*, HTLV-1 or coinfected with HTLV-1 and *S*. *stercoralis*. The Th1 cytokines IFN-γ, TNF-α and IL-12p40 and the Th2 cytokines IL-10 and IL-13 were elevated in mice infected with HTLV-1, *S*. *stercoralis* and coinfected mice. The Th2 cytokine IL-5 was only modestly, yet significantly, elevated in mice infected with *S*. *stercoralis* alone and IL-4 was not measurable in any of the infected humanized mice. In the presence of HTLV-1 and *S*. *stercoralis* the increased IL-5 levels were abated which is consistent with that seen in human coinfections [57].

Humanized mice have been shown to develop a limited human cytokine profile in response to specific stimuli [58,59]. To eliminate any variability between the human cytokine responses that occur between different stem cell donors, all the cohorts of humanized mice were intermixed. Several of the human cytokine levels measured in the serum of the humanized mice are low when compared to human levels of cytokines [60], but they are within the spectrum that has been demonstrated by other humanized mouse experiments [58,59]. These experimental systems are not a complete representation of the human immune response cytokine profiles or cellular responses, but they do provide a starting point to start dissecting the human immune response to pathogens that have no other viable animal model. Comparing human cytokine responses between groups of mice at 7-weeks post infection revealed that humanized mice infected with HTLV-1 or a coinfection of HTLV-1 and *S*. *stercoralis* had significantly higher IFN-γ levels than that seen in mice only infected with *S*. *stercoralis*. Interestingly, TNF-α levels were significantly enhanced in coinfected animals when compared to levels in mice infected with either *S*. *stercoralis* or HTLV-1. TNF-α may play a role in the control of *S*. *strongyloides* infection based on the observation that TNF-α blockers have been reported to induce hyperinfection in patients infected with *S*. *stercoralis* [61]. TNF-α blockers have variable effects on the course of HTLV-1 infection and the development of ATLL [62–65].

Patients coinfected with *S*. *stercoralis* and HTLV-1 exhibit lower levels of TNF-α, IFN-γ, and an overall increase in the frequency of IFN-γ expressing CD8^+^ and CD4^+^ T-cells compared to patients infected solely with HTLV-1 [23,57]. These findings show that the helminth infection can downregulate the exaggerated pro-inflammatory response observed in HTLV-1 infection [20]. In the current study, using humanized mice, we observed increased levels of TNF-α in mice with HTLV-1/*S*. *stercoralis* coinfection. This increase may have been the result of the limited Th2 response induced by *S*. *stercoralis*. It is important to recognize that most of the reported human cases of coinfection with HTLV-1 and *S*. *stercoralis* appear to be the result of chronic infections whereas the infections analyzed in this study were acute infections. It is therefore unlikely that there will be harmony between the presentation of the chronic infections in humans and the acute infections in the humanized mice. Furthermore, study of the coinfections in humanized mice has allowed for the first time the observation of the initial interaction between these two pathogens in a human immune milieu.

Patients with HTLV-1 and *S*. *stercoralis* coinfection have higher parasite burdens and increased proportions of regulatory T-cells [24]. Circulating PBMC were analyzed by flow cytometry to determine the composition of the T-cell compartment. Infection of humanized mice with HTLV-1 significantly increased the number of human hematopoietic cells in circulation. No significant changes in the overall percentages of circulating CD3^+^ and CD4^+^ T-cells were observed, suggesting that they increased proportionately with other hematopoietic cells. CD4^+^CD25^+^ positive T-cells were significantly increased in frequency in both the HTLV-1 infection and HTLV-1/*S*. *stercoralis* coinfection when compared to *S*. *stercoralis* infection alone. The increase in CD4^+^CD25^+^ positive T-cells, which are presumably regulatory T-cells, aligns with observations in humans coinfected with HTLV-1 and *S*. *stercoralis* [24].

The enhanced levels of CD4^+^CD25^+^ positive T-cells may be the result of clonal proliferation, or the development of ATLL or a regulation of the Th1 response [66]. To explore the clonality of the T-cell population, the peripheral blood of the humanized mice was analyzed by TCR-PCR. This PCR based assay cannot differentiate between clonal expansion of T-cells and the development of ATLL but can rule out a generic expansion of regulatory T-cells in response to varying cytokine environments. Analysis of the T-cell recombinations did not find any evidence of clonal proliferation. The increased levels of CD4^+^CD25^+^ T-cells may simply be a response to alterations in the cytokine environment in mice caused by the coinfections. This finding does not preclude the future development of an ATLL-like progression, as was seen in the CD133^+^ humanized NRG mice at later stages of infection [30]. It is likely that early death seen in our model decreases the chance that expansion of certain T-cell clones had adequate time to progress [31].

NSG mice infected with *S*. *stercoralis* and treated with MPA develop a lethal hyperinfection, characterized by a significant increase in numbers of all stages of the parasite and the development of L3a [4]. Similar results were seen in the current study using humanized NSG mice. Coinfection of humanized NSG mice with HTLV-1 and *S*. *stercoralis* did not result in an increase in the number of parasites or the development of L3a. Interestingly, L3i recovered from MPA treated animals were significantly longer than L3i recovered from untreated single infections in humanized mice. L3i recovered from coinfected animals were in two populations, one with the same length as L3i recovered from humanized NSG mice infected with only *S*. *stercoralis* and the other group of L3i the same length as seen in mice treated with MPA. Enhanced nematode growth is associated with the immune status of the host [67–70] and MPA and HTLV-1 may enhance the growth of *S*. *stercoralis* L3i through a shared immune-based mechanism. It has been hypothesized that MPA is effective at enhancing L3i growth through an alteration in the host immune response, which results in the develpment of hyperinfection. Infection with HTLV-1 induces equivalent enhanced growth in some L3i, yet not in others. MPA may induce a systemic response that enhances the growth of most of the L3i, whereas infection with HTLV-1 may induce a localized response that enhances the growth of L3i located in specific microenvironments of the host. The relative ability between MPA and HTLV-1 to enhance L3i growth may help explain why L3a are seen in mice treated with MPA but not in mice infected with HTLV-1. It is possible that hyperinfection would develop in mice coinfected with HTLV-1 and *S*. *stercoralis* if the infections were allowed to persist for extended time periods.

In conclusion, humanized NSG mice are susceptible to coinfection with the human pathogens HTLV-1 and *S*. *stercoralis*. There was an increased level of mortality in the HTLV-1 and coinfected animals when compared to the *S*. *stercoralis* infected individuals. The enhanced mortality was not correlated with either the proviral loads or total viral RNA in the infected animals. Changes in the cytokine profiles in the HTLV-1 infected animals showed a distinct shift towards Th1 similar to the progression of human disease etiology, and Th2 cytokines were elevated in the *S*. *stercoralis* infected mice. Coinfection of humanized mice resulted in elevated TNF-α levels. The CD4^+^CD25^+^ T-cell population was significantly expanded in the HTLV-1 and coinfected groups, although this expansion was not clonal in nature. Worm counts in the coinfection group were not different from the *S*. *stercoralis* infected animals and they did not have any L3a in the L3 populations. There was, however, an enhancement in the lengths of the L3i recovered from the coinfection group, which suggest that there are interactions occurring between the two pathogens affecting worm development as seen in the MPA treated animals. Thus, humanized NSG mice provide a robust model to study the interactions that occur between HTLV-1, *S*. *stercoralis* and the immune system *in vivo*. Further development of this model with humanized mice that have been engineered to produce strong Th2 responses may further enhance the translational aspects of this model.

## References

[1] Bisoffi Z, Buonfrate D, Montresor A, Requena-Mendez A, Munoz J, Krolewiecki AJ, et al. Strongyloides stercoralis: a plea for action. PLoS Negl Trop Dis. 2013;7[5]:e2214. doi: 10.1371/journal.pntd.0002214 23675546 PMC3649953

[2] Schar F, Trostdorf U, Giardina F, Khieu V, Muth S, Marti H, et al. Strongyloides stercoralis: Global Distribution and Risk Factors. PLoS Negl Trop Dis. 2013;7[7]:e2288. doi: 10.1371/journal.pntd.0002288 23875033 PMC3708837

[3] Ganesh S, Cruz RJ. Strongyloidiasis. Gastroenterol Hepatol [N Y]. 2011;7[3]:194–6. 21528049 PMC3079152

[4] Patton JB, Bonne-Année S, Deckman J, Hess JA, Torigian A, Nolan TJ, et al. Methylprednisolone acetate induces, and Δ7-dafachronic acid suppresses, Strongyloides stercoralis hyperinfection in NSG mice. Proceedings of the National Academy of Sciences of the United States of America. 2018;115[1]:204–9. doi: 10.1073/pnas.1712235114 29203662 PMC5776800

[5] Greaves D, Coggle S, Pollard C, Aliyu SH, Moore EM. Strongyloides stercoralis infection. BMJ. 2013;347:f4610. doi: 10.1136/bmj.f4610 23900531

[6] Vasquez-Rios G, Pineda-Reyes R, Pineda-Reyes J, Marin R, Ruiz EF, Terashima A. Strongyloides stercoralis hyperinfection syndrome: a deeper understanding of a neglected disease. J Parasit Dis. 2019;43[2]:167–75. doi: 10.1007/s12639-019-01090-x 31263320 PMC6570730

[7] Krolewiecki A, Nutman TB. Strongyloidiasis: A Neglected Tropical Disease. Infect Dis Clin North Am. 2019;33[1]:135–51. doi: 10.1016/j.idc.2018.10.006 30712758 PMC6367705

[8] Gessain A, Cassar O. Epidemiological Aspects and World Distribution of HTLV-1 Infection. Frontiers in microbiology. 2012;3:388. doi: 10.3389/fmicb.2012.00388 23162541 PMC3498738

[9] Proietti FA, Carneiro-Proietti ABF, Catalan-Soares BC, Murphy EL. Global epidemiology of HTLV-I infection and associated diseases. Oncogene. 2005;24[39]:6058–68. doi: 10.1038/sj.onc.1208968 16155612

[10] Araya N, Sato T, Yagishita N, Ando H, Utsunomiya A, Jacobson S, et al. Human T-Lymphotropic Virus Type 1 [HTLV-1] and Regulatory T Cells in HTLV-1-Associated Neuroinflammatory Disease. Viruses. 2011;3[9]:1532–48. doi: 10.3390/v3091532 21994794 PMC3187691

[11] Malpica L, White AC Jr., Leguia C, Freundt N, Barros N, Chian C, et al. Regulatory T cells and IgE expression in duodenal mucosa of Strongyloides stercoralis and human T lymphotropic virus type 1 co-infected patients. PLoS Negl Trop Dis. 2019;13[6]:e0007415. doi: 10.1371/journal.pntd.0007415 31170141 PMC6581271

[12] Hoces D, Barros N, Woll F, Bauer A, White AC Jr., Montes M. Regulatory T cell expansion resolves after effective strongyloidiasis treatment in subjects with HTLV-1 co-infection. Parasitol Int. 2020;76:102092. doi: 10.1016/j.parint.2020.102092 32120049

[13] Gonçalves DU, Proietti FA, Ribas JGR, Araújo MG, Pinheiro SR, Guedes AC, et al. Epidemiology, treatment, and prevention of human T-cell leukemia virus type 1-associated diseases. Clin Microbiol Rev. 2010;23[3]:577–89. doi: 10.1128/CMR.00063-09 20610824 PMC2901658

[14] Bangham CRM, Cook LB, Melamed A. HTLV-1 clonality in adult T-cell leukaemia and non-malignant HTLV-1 infection. Seminars in Cancer Biology. 2014;26[100]:89–98. doi: 10.1016/j.semcancer.2013.11.003 24316494 PMC4062949

[15] Futsch N, Mahieux R, Dutartre H. HTLV-1, the Other Pathogenic Yet Neglected Human Retrovirus: From Transmission to Therapeutic Treatment. Viruses. 2017;10[1]. doi: 10.3390/v10010001 29267225 PMC5795414

[16] Reese TA, Wakeman BS, Choi HS, Hufford MM, Huang SC, Zhang X, et al. Helminth infection reactivates latent gamma-herpesvirus via cytokine competition at a viral promoter. Science. 2014;345[6196]:573–7. doi: 10.1126/science.1254517 24968940 PMC4531374

[17] Osborne LC, Monticelli LA, Nice TJ, Sutherland TE, Siracusa MC, Hepworth MR, et al. Coinfection. Virus-helminth coinfection reveals a microbiota-independent mechanism of immunomodulation. Science. 2014;345[6196]:578–82. doi: 10.1126/science.1256942 25082704 PMC4548887

[18] Stewart DM, Ramanathan R, Mahanty S, Fedorko DP, Janik JE, Morris JC. Disseminated Strongyloides stercoralis Infection in HTLV-1-Associated Adult T-Cell Leukemia/Lymphoma. Acta Haematologica. 2011;126[2]:63–7. doi: 10.1159/000324799 21474923 PMC3080579

[19] Carvalho EM, Da Fonseca Porto A. Epidemiological and clinical interaction between HTLV-1 and Strongyloides stercoralis. Parasite Immunology. 2004;26[11–12]:487–97. doi: 10.1111/j.0141-9838.2004.00726.x 15771684

[20] Porto AF, Neva FA, Bittencourt H, Lisboa W, Thompson R, Alcântara L, et al. HTLV-1 decreases Th2 type of immune response in patients with strongyloidiasis. Parasite Immunology. 2001;23[9]:503–7. doi: 10.1046/j.1365-3024.2001.00407.x 11589779

[21] Dykie A, Wijesinghe T, Rabson AB, Madugula K, Farinas C, Wilson S, et al. Human T-cell Leukemia Virus Type 1 and Strongyloides stercoralis: Partners in Pathogenesis. Pathogens. 2020;9[11]. doi: 10.3390/pathogens9110904 33137906 PMC7692131

[22] Morel PA, Oriss TB. Crossregulation between Th1 and Th2 cells. Crit Rev Immunol. 1998;18[4]:275–303. doi: 10.1615/critrevimmunol.v18.i4.10 9704191

[23] Porto AF, Santos SB, Muniz AL, Basilio V, Rodrigues W, Neva FA, et al. Helminthic infection down-regulates type 1 immune responses in human T cell lymphotropic virus type 1 [HTLV-1] carriers and is more prevalent in HTLV-1 carriers than in patients with HTLV-1-associated myelopathy/tropical spastic paraparesis. J Infect Dis. 2005;191[4]:612–8. doi: 10.1086/427560 15655786

[24] Montes M, Sanchez C, Verdonck K, Lake JE, Gonzalez E, Lopez G, et al. Regulatory T Cell Expansion in HTLV-1 and Strongyloidiasis Co-infection Is Associated with Reduced IL-5 Responses to Strongyloides stercoralis Antigen. PLoS Neglected Tropical Diseases. 2009;3[6]. doi: 10.1371/journal.pntd.0000456 19513105 PMC2686100

[25] Satoh M, Toma H, Sugahara K, Etoh K, Shiroma Y, Kiyuna S, et al. Involvement of IL-2/IL-2R system activation by parasite antigen in polyclonal expansion of CD4[+]25[+] HTLV-1-infected T-cells in human carriers of both HTLV-1 and S. stercoralis. Oncogene. 2002;21[16]:2466–75. doi: 10.1038/sj.onc.1205329 11971181

[26] Gabet A-S, Mortreux F, Talarmin A, Plumelle Y, Leclercq I, Leroy A, et al. High circulating proviral load with oligoclonal expansion of HTLV-1 bearing T cells in HTLV-1 carriers with strongyloidiasis. Oncogene. 2000;19[43]:4954–60. doi: 10.1038/sj.onc.1203870 11042682

[27] Gillet NA, Cook L, Laydon DJ, Hlela C, Verdonck K, Alvarez C, et al. Strongyloidiasis and infective dermatitis alter human T lymphotropic virus-1 clonality in vivo. PLoS Pathog. 2013;9[4]:e1003263. doi: 10.1371/journal.ppat.1003263 23592987 PMC3617147

[28] Ginwala R, Caruso B, Khan ZK, Pattekar A, Chew GM, Corley MJ, et al. HTLV-1 Infection and Neuropathogenesis in the Context of Rag1-/-γc-/- [RAG1-Hu] and BLT Mice. J Neuroimmune Pharmacol. 2017;12[3]:504–20. doi: 10.1007/s11481-017-9740-y 28374110 PMC5529230

[29] Miyazato P, Yasunaga J, Taniguchi Y, Koyanagi Y, Mitsuya H, Matsuoka M. De novo human T-cell leukemia virus type 1 infection of human lymphocytes in NOD-SCID, common gamma-chain knockout mice. J Virol. 2006;80[21]:10683–91. doi: 10.1128/JVI.01009-06 16943297 PMC1641804

[30] Tezuka K, Xun R, Tei M, Ueno T, Tanaka M, Takenouchi N, et al. An animal model of adult T-cell leukemia: humanized mice with HTLV-1-specific immunity. Blood. 2014;123[3]:346–55. doi: 10.1182/blood-2013-06-508861 24196073

[31] Galli V, Nixon CC, Strbo N, Artesi M, de Castro-Amarante MF, McKinnon K, et al. Essential Role of HTLV-1 orf-I in Lethal Proliferation of CD4[+] Cells in Humanized Mice. J Virol. 2019. doi: 10.1128/JVI.00565-19 31315992 PMC6744231

[32] Shultz LD, Lyons BL, Burzenski LM, Gott B, Chen X, Chaleff S, et al. Human lymphoid and myeloid cell development in NOD/LtSz-scid IL2R gamma null mice engrafted with mobilized human hemopoietic stem cells. J Immunol. 2005;174[10]:6477–89. doi: 10.4049/jimmunol.174.10.6477 15879151

[33] Miyoshi I, Kubonishi I, Yoshimoto S, Shiraishi Y. A T-cell line derived from normal human cord leukocytes by co-culturing with human leukemic T-cells. Gan. 1981;72[6]:978–81. 6281119

[34] Schad GA, Hellman ME, Muncey DW. Strongyloides stercoralis: hyperinfection in immunosuppressed dogs. Exp Parasitol. 1984;57[3]:287–96. doi: 10.1016/0014-4894(84)90103-6 6723900

[35] Trainor KJ, Brisco MJ, Wan JH, Neoh S, Grist S, Morley AA. Gene rearrangement in B- and T-lymphoproliferative disease detected by the polymerase chain reaction. Blood. 1991;78[1]:192–6. 1648975

[36] Team RC. R: A Language and Enviroment for Statistical Computing. In: Computing RFfS, editor. Vienna, Austria 2017.

[37] Pinheiro J BD, DebRoy S, Sarkar D, R Core Team. Linear and Nonlinear Mixed Effects Models. R package version 3.1–144 ed2020.

[38] Hothorn T, Bretz F, Westfall P. Simultaneous inference in general parametric models. Biom J. 2008;50[3]:346–63. doi: 10.1002/bimj.200810425 18481363

[39] Araya N, Sato T, Ando H, Tomaru U, Yoshida M, Coler-Reilly A, et al. HTLV-1 induces a Th1-like state in CD4+CCR4+ T cells. The Journal of clinical investigation. 2014;124[8]:3431–42. doi: 10.1172/JCI75250 24960164 PMC4109535

[40] Yamano Y, Coler-Reilly A. HTLV-1 induces a Th1-like state in CD4[+]CCR4[+] T cells that produces an inflammatory positive feedback loop via astrocytes in HAM/TSP. J Neuroimmunol. 2017;304:51–5. doi: 10.1016/j.jneuroim.2016.08.012 27542993

[41] Anuradha R, Munisankar S, Dolla C, Kumaran P, Nutman TB, Babu S. Parasite Antigen-Specific Regulation of Th1, Th2, and Th17 Responses in Strongyloides stercoralis Infection. J Immunol. 2015;195[5]:2241–50. doi: 10.4049/jimmunol.1500745 26202988 PMC4546867

[42] Hovette P, Tuan JF, Camara P, Lejean Y, Lô N, Colbacchini P. [Pulmonary strongyloidiasis complicated by E. coli meningitis in a HIV-1 and HTLV-1 positive patient]. Presse Medicale [Paris, France: 1983]. 2002;31[22]:1021–3. 12148255

[43] Richter J, Schwarz U, Duwe S, Ellerbrok H, Poggensee G, Pauli G. [Recurrent strongyloidiasis as an indicator of HTLV-1 infection]. Dtsch Med Wochenschr. 2005;130[16]:1007–10. doi: 10.1055/s-2005-866778 15830313

[44] Vadlamudi RS, Chi DS, Krishnaswamy G. Intestinal strongyloidiasis and hyperinfection syndrome. Clin Mol Allergy. 2006;4:8. doi: 10.1186/1476-7961-4-8 16734908 PMC1538622

[45] Shultz LD, Schweitzer PA, Christianson SW, Gott B, Schweitzer IB, Tennent B, et al. Multiple defects in innate and adaptive immunologic function in NOD/LtSz-scid mice. J Immunol. 1995;154[1]:180–91. 7995938

[46] Demontis MA, Sadiq MT, Golz S, Taylor GP. HTLV-1 viral RNA is detected rarely in plasma of HTLV-1 infected subjects. J Med Virol. 2015;87[12]:2130–4. doi: 10.1002/jmv.24264 25982784

[47] Nagai M, Usuku K, Matsumoto W, Kodama D, Takenouchi N, Moritoyo T, et al. Analysis of HTLV-I proviral load in 202 HAM/TSP patients and 243 asymptomatic HTLV-I carriers: high proviral load strongly predisposes to HAM/TSP. J Neurovirol. 1998;4[6]:586–93. doi: 10.3109/13550289809114225 10065900

[48] Plumelle Y, Gonin C, Edouard A, Bucher BJ, Thomas L, Brebion A, et al. Effect of Strongyloides stercoralis infection and eosinophilia on age at onset and prognosis of adult T-cell leukemia. Am J Clin Pathol. 1997;107[1]:81–7. doi: 10.1093/ajcp/107.1.81 8980372

[49] Arisawa K, Soda M, Ono M, Uemura H, Hiyoshi M, Suyama A. Trends of incidence rate of adult T-cell leukemia/lymphoma in an HTLV-1 endemic area in Japan. International journal of cancer. 2009;125[3]:737–8. doi: 10.1002/ijc.24420 19437534

[50] Tajima K, Kuroishi T. Estimation of rate of incidence of ATL among ATLV [HTLV-I] carriers in Kyushu, Japan. Japanese journal of clinical oncology. 1985;15[2]:423–30. 2991627

[51] Kataoka K, Nagata Y, Kitanaka A, Shiraishi Y, Shimamura T, Yasunaga J, et al. Integrated molecular analysis of adult T cell leukemia/lymphoma. Nat Genet. 2015;47[11]:1304–15. doi: 10.1038/ng.3415 26437031

[52] Gillet NA, Malani N, Melamed A, Gormley N, Carter R, Bentley D, et al. The host genomic environment of the provirus determines the abundance of HTLV-1-infected T-cell clones. Blood. 2011;117[11]:3113–22. doi: 10.1182/blood-2010-10-312926 21228324 PMC3062313

[53] Akbarin MM, Rahimi H, HassanNia T, Shoja Razavi G, Sabet F, Shirdel A. Comparison of HTLV-I Proviral Load in Adult T Cell Leukemia/Lymphoma [ATL], HTLV-I-Associated Myelopathy [HAM-TSP] and Healthy Carriers. Iran J Basic Med Sci. 2013;16[3]:208–12. 24470863 PMC3881246

[54] Yamano Y, Nagai M, Brennan M, Mora CA, Soldan SS, Tomaru U, et al. Correlation of human T-cell lymphotropic virus type 1 [HTLV-1] mRNA with proviral DNA load, virus-specific CD8[+] T cells, and disease severity in HTLV-1-associated myelopathy [HAM/TSP]. Blood. 2002;99[1]:88–94. doi: 10.1182/blood.v99.1.88 11756157

[55] Iwanaga M, Watanabe T, Utsunomiya A, Okayama A, Uchimaru K, Koh K-R, et al. Human T-cell leukemia virus type I [HTLV-1] proviral load and disease progression in asymptomatic HTLV-1 carriers: a nationwide prospective study in Japan. Blood. 2010;116[8]:1211–9. doi: 10.1182/blood-2009-12-257410 20448111

[56] Best I, Lopez G, Verdonck K, Gonzalez E, Tipismana M, Gotuzzo E, et al. IFN-gamma production in response to Tax 161–233, and frequency of CD4+ Foxp3+ and Lin HLA-DRhigh CD123+ cells, discriminate HAM/TSP patients from asymptomatic HTLV-1-carriers in a Peruvian population. Immunology. 2009;128[1 Suppl]:e777–86. doi: 10.1111/j.1365-2567.2009.03082.x 19740339 PMC2753904

[57] Salles F, Bacellar A, Amorim M, Orge G, Sundberg M, Lima M, et al. Treatment of strongyloidiasis in HTLV-1 and Strongyloides stercoralis coinfected patients is associated with increased TNFalpha and decreased soluble IL2 receptor levels. Trans R Soc Trop Med Hyg. 2013;107[8]:526–9. doi: 10.1093/trstmh/trt052 23843560 PMC3735360

[58] Skirecki T, Drechsler S, Hoser G, Jafarmadar M, Siennicka K, Pojda Z, et al. The Fluctuations of Leukocytes and Circulating Cytokines in Septic Humanized Mice Vary With Outcome. Front Immunol. 2019;10:1427. doi: 10.3389/fimmu.2019.01427 31297113 PMC6607920

[59] Unsinger J, McDonough JS, Shultz LD, Ferguson TA, Hotchkiss RS. Sepsis-induced human lymphocyte apoptosis and cytokine production in "humanized" mice. J Leukoc Biol. 2009;86[2]:219–27. doi: 10.1189/jlb.1008615 19369639 PMC2726769

[60] Kleiner G, Marcuzzi A, Zanin V, Monasta L, Zauli G. Cytokine levels in the serum of healthy subjects. Mediators Inflamm. 2013;2013:434010. doi: 10.1155/2013/434010 23533306 PMC3606775

[61] Krishnamurthy R, Dincer HE, Whittemore D. Strongyloides stercoralis hyperinfection in a patient with rheumatoid arthritis after anti-TNF-alpha therapy. Journal of clinical rheumatology: practical reports on rheumatic & musculoskeletal diseases. 2007;13[3]:150–2. 17551383 10.1097/RHU.0b013e3180690933

[62] Fukui S, Nakamura H, Takahashi Y, Iwamoto N, Hasegawa H, Yanagihara K, et al. Tumor necrosis factor alpha inhibitors have no effect on a human T-lymphotropic virus type-I [HTLV-I]-infected cell line from patients with HTLV-I-associated myelopathy. BMC immunology. 2017;18[1]:7. doi: 10.1186/s12865-017-0191-2 28158970 PMC5292003

[63] Bittencourt AL, Oliveira PD, Bittencourt VG, Carvalho EM, Farre L. Adult T-cell leukemia/lymphoma triggered by adalimumab. J Clin Virol. 2013;58[2]:494–6. doi: 10.1016/j.jcv.2013.07.011 23911677

[64] Frenzel L, Moura B, Marcais A, Chapdelaine H, Hermine O. HTLV-1-associated arthropathy treated with anti-TNF-alpha agent. Joint Bone Spine. 2014;81[4]:360–1. doi: 10.1016/j.jbspin.2013.10.006 24289962

[65] Umekita K, Umeki K, Miyauchi S, Ueno S, Kubo K, Kusumoto N, et al. Use of anti-tumor necrosis factor biologics in the treatment of rheumatoid arthritis does not change human T-lymphotropic virus type 1 markers: a case series. Mod Rheumatol. 2015;25[5]:794–7. doi: 10.3109/14397595.2013.844389 24252020

[66] Satou Y, Utsunomiya A, Tanabe J, Nakagawa M, Nosaka K, Matsuoka M. HTLV-1 modulates the frequency and phenotype of FoxP3+CD4+ T cells in virus-infected individuals. Retrovirology. 2012;9:46. doi: 10.1186/1742-4690-9-46 22647666 PMC3403885

[67] Gebreselassie NG, Moorhead AR, Fabre V, Gagliardo LF, Lee NA, Lee JJ, et al. Eosinophils preserve parasitic nematode larvae by regulating local immunity. J Immunol. 2012;188[1]:417–25. doi: 10.4049/jimmunol.1101980 22131328 PMC3244516

[68] Riner DK, Ferragine CE, Maynard SK, Davies SJ. Regulation of innate responses during pre-patent schistosome infection provides an immune environment permissive for parasite development. PLoS Pathog. 2013;9[10]:e1003708. doi: 10.1371/journal.ppat.1003708 24130499 PMC3795041

[69] Lamb EW, Walls CD, Pesce JT, Riner DK, Maynard SK, Crow ET, et al. Blood fluke exploitation of non-cognate CD4+ T cell help to facilitate parasite development. PLoS Pathog. 2010;6[4]:e1000892. doi: 10.1371/journal.ppat.1000892 20442785 PMC2861709

[70] Babayan SA, Read AF, Lawrence RA, Bain O, Allen JE. Filarial parasites develop faster and reproduce earlier in response to host immune effectors that determine filarial life expectancy. PLoS Biol. 2010;8[10]:e1000525. doi: 10.1371/journal.pbio.1000525 20976099 PMC2957396

